# Physical Activity Behavior and Its Association With Global Cognitive Function Three Months After Stroke: The Nor-COAST Study^†^

**DOI:** 10.1093/ptj/pzad092

**Published:** 2023-07-13

**Authors:** Geske Luzum, Mari Gunnes, Stian Lydersen, Ingvild Saltvedt, Xiangchun Tan, Pernille Thingstad, Gyrd Thrane, Torunn Askim

**Affiliations:** Department of Neuromedicine and Movement Science, Faculty of Medicine and Health Science, NTNU-Norwegian University of Science and Technology, Trondheim, Norway; Department of Health Research, SINTEF, Trondheim, Norway; Department of Mental Health, Faculty of Medicine and Health Sciences, NTNU-Norwegian University of Science and Technology, Trondheim, Norway; Department of Neuromedicine and Movement Science, Faculty of Medicine and Health Science, NTNU-Norwegian University of Science and Technology, Trondheim, Norway; Department of Geriatric Medicine, St. Olavs hospital, Trondheim University Hospital, Trondheim, Norway; Department of Neuromedicine and Movement Science, Faculty of Medicine and Health Science, NTNU-Norwegian University of Science and Technology, Trondheim, Norway; Department of Neuromedicine and Movement Science, Faculty of Medicine and Health Science, NTNU-Norwegian University of Science and Technology, Trondheim, Norway; Department of Health and Welfare Services, City of Trondheim, Trondheim, Norway; Department of Health and Care Science, Faculty of Health, The Arctic University of Norway, Tromsø, Norway; Department of Neuromedicine and Movement Science, Faculty of Medicine and Health Science, NTNU-Norwegian University of Science and Technology, Trondheim, Norway

**Keywords:** Adherence, Cognitive Function, Physical Activity, Physical Capacity, Stroke

## Abstract

**Objective:**

The purposes of this study were to determine the association between physical activity (PA) behavior and global cognitive function 3 months after stroke and to explore the role of physical capacity as a mediating factor.

**Methods:**

Participants with stroke were successively recruited at 5 different hospitals in Norway. PA was measured using accelerometers, with a follow-up period of 7 consecutive days, and global cognitive function was assessed using the Montreal Cognitive Assessment (MoCA). The general pattern of PA and the percentage of participants adhering to World Health Organization PA recommendations (at least 150 minutes of moderate-intensity aerobic PA per week) were investigated using descriptive statistics. Multiple regression and mediator analyses were used to examine the relationship between PA behavior and MoCA scores; physical capacity, measured with the Short Physical Performance Battery, served as the mediating variable.

**Results:**

A total of 193 women (42.6%) and 260 men (57.4%) with a median age of 73.7 years (25th and 75th percentiles = 65.8 and 80.4, respectively) and a median MoCA score of 25 points (25th and 75th percentiles = 22 and 27, respectively) were included. Mean total time spent walking at moderate intensity was 251.7 (SD = 164.6) min/wk (mean bout length = 20.9 [SD = 7.3] seconds), which indicated 69.3% adherence to World Health Organization guidelines. With each point decrease in the MoCA score, there was an expected 8.6% increase in the odds of nonadherence to PA recommendations. Physical capacity was identified as an important mediating factor, explaining the strength of the association between cognition and PA behavior.

**Conclusions:**

In contrast to previous research, in the present study, most participants adhered to the updated global PA guidelines. However, people who had survived stroke and had reduced cognitive function were at higher risk of inactivity, an association mediated by physical capacity.

**Impact:**

A better understanding of the association between cognition and PA behavior after stroke might help for developing more targeted early-onset interventions.

## Introduction

Stroke is considered to be the second leading cause of death and the third leading cause of disability-adjusted life years worldwide.^1^ The prevalence of cognitive impairment after stroke has been estimated at approximately 53%, based on the diagnostic approach used.^2^^,^^3^ The manifestations of cognitive impairment in activities of daily living vary, depending on which domains of cognitive function are affected.^4–6^ Besides its association with increased dependency, cognitive impairment has been linked to reduced quality of life and accelerated functional decline,^7–9^ with decreased gait speed and walking capacity.^10^ As yet, there is no cure for cognitive impairment, despite growing evidence on factors that may play a role in primary and secondary prevention. It appears that physical activity (PA), an essential part of stroke care, is associated with improving and/or preserving cognitive function^11^^,^^12^; underlying mechanisms are linked to increased cerebral oxygen supply and cerebral perfusion, which stimulate neurogenesis, angiogenesis, and synaptic plasticity in the brain. A reduction of inflammatory processes is further indicated through an increase of neurotrophic factors, reduced oxidative stress and a reduction of β-amyloid formation.^13^^,^^14^

Despite growing knowledge of the benefits of PA, it has been estimated that only 1 of 4 adults worldwide achieves the recommended goal of at least 150 minutes of moderate to vigorous PA per week.^15^ PA levels among stroke survivors have been shown to be significantly lower than in a healthy reference population, on average achieving half the daily step counts and having fitness levels well below the average for their age.^16–18^ Consequently, it can be assumed that the rate of adherence to PA recommendations is even lower among the population of stroke survivors.^19^ As poor adherence to PA recommendations influences the effectiveness of rehabilitative and preventive interventions,^20^^,^^21^ early identification of individuals at risk of inactivity and subsequent implementation of adherence-enhancing targeted interventions^20^ will remain a crucial task for physical therapists in the near future.

Evidence from longitudinal studies indicates that a decrease in cognitive function after stroke may represent a risk factor for inactivity.^22^^,^^23^ Tentative theories to explain the reciprocal connection establish cognition as the ability to overcome barriers to PA as well as to understand the importance of PA for future health outcomes.^17^ Individuals with greater cognitive impairment may have less awareness around the amount of time they have spent in a sedentary position or may require greater assistance or supervision when walking around their home or community.^24^ Additionally, the close interactions between cognitive and physical domains in older adults^25^^,^^26^ and stroke survivors^27^ may play an important role in the association between cognitive function and PA behavior. Heterogeneity regarding physical factors among study populations may be an explanation why others^16^^,^^28^ did not observe an association between cognition and PA. Yet, no studies have established whether the relationship between global cognitive function and PA behavior may be explained by the association between cognitive function and physical capacity; and there are only small studies with inconsistent findings^18^^,^^23^^,^^29^ that have objectively assessed the association between habitual, free-living PA and cognitive function after stroke. Therefore, the overarching aim of this study was to describe PA behavior 3 months after stroke and its relationship with global cognitive function. The 3 specific aims of this study were to objectively determine PA behavior in terms of amount, intensity, and frequency of PA and adherence to World Health Organization (WHO) PA guidelines 3 months after stroke; to investigate whether global cognitive function is associated with PA behavior and adherence to WHO guidelines; and to explore whether the association of cognitive function, PA behavior, and adherence to PA guidelines is mediated through physical capacity.

## Methods

This was a cross-sectional observational study based on data from the prospective multicenter Norwegian Cognitive Impairment After Stroke study (Nor-COAST). Nor-COAST seeks to determine cognitive impairment levels in the general Norwegian stroke population as well as to identify biological and clinical markers associated with cognitive impairments following stroke. The detailed primary study design of Nor-COAST has been described previously.^30^

Recruitment and testing were conducted at 3 university hospitals and 2 local hospitals, by 3 different Norwegian health authorities. All admitted patients were screened for eligibility. Eligible patients were included if they were able and willing to sign an informed consent form; patients who were not able to give informed consent were also included if their next of kin gave verbal consent for participation, in keeping with national consent procedures for patients who are unable to consent for themselves. Patients (≥18 years) with a clinical diagnosis of stroke^31^ who were admitted to a hospital within 7 days after symptom debut were successively recruited from May 2015 to March 2017 with final follow up in March 2020. Participants needed to be able to speak a Scandinavian language and to have an expected survival of more than 3 months. In addition to the Nor-COAST inclusion criteria, people with erroneous accelerometer recordings, with fewer than 4 days of accelerometer recordings, or with missing data on global cognitive function at a 3-month follow-up point were excluded from this study.

Information on sociodemographic and personal data, including relevant health data, was obtained only during baseline examinations, using medical records and standardized questionnaires with patients and/or caregivers. Severity of stroke was quantified using the National Institutes of Health Stroke Scale (NIHSS); the maximum possible score is 42 and 0 indicates no symptoms after stroke.^6^

Data collected at the 3-month follow-up point included functional and behavioral outcomes. Global disability after stroke was obtained using the modified Rankin Scale^32^; scores between 0 and 6 indicate the level of dependency in daily living, with 6 referring to death. Global cognitive function was assessed using the Montreal Cognitive Assessment (MoCA); the MoCA total score ranges from 0 to 30, with values lower than 26 indicating a poststroke cognitive disorder. We added 1 point to the total score if participants had ≤12 years of education.^33^ Individuals with scores of 26 to 30 were interpreted as having normal functioning.^34^ MoCA relies on verbal responses; testing therefore precludes individuals with trouble speaking or understanding other people speaking (eg, aphasia). We evaluated physical capacity of the lower extremity using the Short Physical Performance Battery (SPPB). The performance-based SPPB evaluates balance, gait speed, and functional strength, with each subtask being graded on a 4-point scale.^35^ Participants were divided into high-functioning (10–12 points), medium-functioning (7–9 points), and low-functioning (0–6 points) groups, in accordance with their total SPPB scores.^36^

PA behaviors were measured using triaxial activPAL accelerometers (PAL Technologies Ltd, Glasgow, UK).^37^ The sensor was attached to the front of the unaffected thigh of each participant, in line with a 24-hour wearing protocol, with a follow-up period of 7 consecutive days. activPAL sensors have been shown to be accurate and reliable in estimating free-living PA and are also a valid tool for objectively measuring PA behavior (time spent in standing, walking, and sitting/lying position) in the people with stroke.^38^^,^^39^ Devices were configured with a sampling frequency of 20 Hz, as recommended by the manufacturer. Intensity of PA was quantified in absolute terms using the metabolic equivalent of task (MET), which was calculated using the activPAL proprietary algorithm and which classified activities into light (1.5 to <3 METs), moderate (3 to <6 METs), and vigorous (≥6 METs) intensities.

Visual side-by-side examination using PALanalysis V8 software (PAL Technologies) was conducted for quality control and for validation of all data records, including the detection of nonwear time. Daytime activity was defined as any activity recognized in the period from 8:00 am to 11:30 pm. The fixed time window was obtained from the central tendency of the wake–sleep pattern (wake-up time and bedtime), deduced from visual inspection of the PALanalysis output using random sampling involving participants from each of the recruiting hospitals. PA data was processed using MATLAB software (R2021a; The MathWorks, Inc, Natick, MA, USA); outcomes which consisted of any upright activities during daytime, were extracted. Filter and segmentation rules were based on the standard algorithm of the manufacturer but adjusted for the present population (VANE algorithm; PAL Technologies). Sedentary events were established with a minimum length of 10 seconds, adopting the default settings, while standing and walking events were established with a minimum length of 3 seconds.^40^ For comprehensive examination of the patterns of time spent being physically active, the cumulative bouts spent in upright behaviors or walking were assigned to 6 predefined zones: zone 1 (3 seconds to <5 minutes) and zone 2 (5 minutes to <10 minutes) were defined as short-bout PA, while zone 3 (10 minutes to <15 minutes), zone 4 (15 minutes to <20 minutes), zone 5 (20 minutes to <30 minutes), and zone 6 (≥30 minutes) were defined as long-bout PA.

### Data Analysis

Independent-samples *t* test, Mann–Whitney *U* test, and Pearson χ-square test were used to assess group differences in the baseline characteristics of people who were included and people who were not included in order to assess the potential for selection bias (Suppl. Tab. 1). Descriptive data are presented as mean and SD or as count percentage. We present the median and interquartile range (25th and 75th percentiles) as well if continuous data had a nonnormal distribution (visual inspection of Q-Q plots). Where applicable, we present effect measures as regression coefficients and the odds ratio with 95% CI.

To address aim 1 of this study, 5 continuous variables were extracted from the data to quantify PA behavior: time spent in moderate PA (MPA) walking (min/wk); time spent in light PA (LPA) walking (min/wk); time spent in vigorous PA walking (min/wk); time spent upright (upright time) (min/wk); and number of sit-to-stand transitions per week. Duration estimates were both presented zone-wise and as mean total per week. Additionally, 1 dichotomous variable, adherence to WHO PA recommendations,^41^ was calculated; for this calculation, an accumulation of ≥150 minutes of MPA per week was defined as adherence to the recommendations.

To address aims 2 and 3, multiple regression analyses and logistic regression analyses were performed for continuous outcome variables and dichotomous outcome variables, respectively. Normal distribution of residuals was assessed visually using Q-Q plots. Sex, age, and education served as confounding variables in all regression analyses. In a subsequent step, a mediation analysis was conducted to explore whether the association of cognition with PA behavior operates through the mediating variable of physical capacity (SPPB total score). Direct and indirect effects of global cognitive function and adherence to PA recommendations were estimated using logistic regression–based mediation analysis. The term direct effect refers to the specific relationship between the independent variable and the dependent variable that is not explained by the mediator variable. Indirect effect refers to the relationship between the independent variable and the dependent variable that is explained by 1 or more mediator variables. We further use the term total effect, including both direct and indirect effects. Log-odds metrics were transformed into odds ratio metrics for better interpretation. All mediation analyses were performed independent of an existing predetermined statistically significant association of MoCA and PA behavior variables.^42^ The *P* value for the indirect effect of adherence to WHO recommendations was derived from the Sobel Test.^42^ Confidence intervals in the mediation analyses were derived from bootstrapping using 5000 bootstrap samples. Mediation analyses were performed using PROCESS procedure version 4.0.^42^ Because of multiple hypotheses, 2-sided *P* values of ≤.01 were considered statistically significant. All statistical analyses were performed using SPSS (IBM SPSS Statistics for Windows, Version 26.0; IBM Corp, Armonk, NY, USA).

### Role of the Funding Source

The funders played no role in the design, conduct, or reporting of this study.

## Results

**Figure 1:**
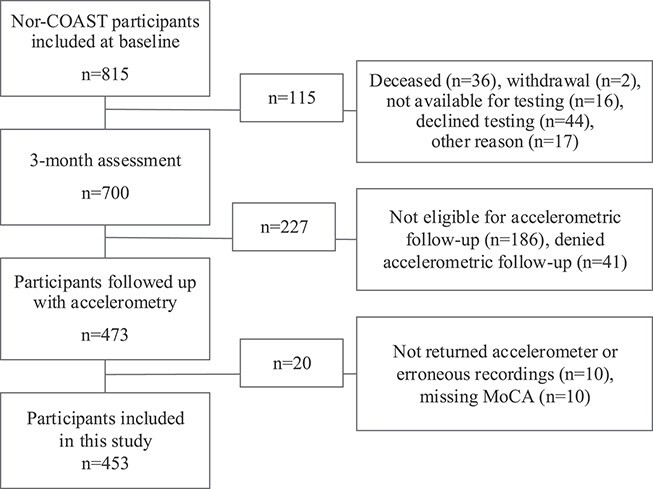
Flowchart for inclusion of participants. MoCA = Montreal Cognitive Assessment; Nor-COAST = prospective multicenter Norwegian Cognitive Impairment After Stroke study.

Figure 1 shows the stream of participants in this study and the reasons for exclusion. After participants without valid recordings or missing data in the MoCA were excluded, the final study population consisted of 453 participants. Table 1 shows participant characteristics, including functional assessments. We found a higher age and higher proportion of living alone among people who were not participants (Suppl. Tab. 1).

**Table 1:** Characteristics of Participants*^a^*

**Characteristic**	**No. of Participants**	**Value**
Age, y	453	
Mean (SD)		72.49 (11.31)
Median (25th and 75th percentiles)		73.65 (65.83 and 80.39)
Sex, no. (%) men	453	260 (57.4)
Ethnicity, no. (%) White	453	447 (98.68)
Education, y, mean (SD)	453	12.43 (3.64)
Socioeconomic status, no. (%)	452	
Working		101 (22.35)
Retired		330 (73.01)
On sick leave		2 (0.44)
Receiving disability pension		15 (3.32)
Other		4 (0.88)
Living alone, no. (%)	453	143 (31.6)
BMI, kg/m^2^, mean (SD)	453	26.14 (4.25)
Diabetes, no. (%)	447	81 (18.12)
Prior CVD, no. (%)	453	99 (21.85)
Type of stroke, no. (%)	453	
Ischemic		416 (91.8)
Hemorrhagic		37 (8.2)
NIHSS score at admission, no. (%)	442	
<3		225 (50.9)
3–5		126 (28.51)
6–10		51 (11.54)
>10		40 (9.05)
Modified Rankin Scale score, no. (%)	452	
0		70 (15.5)
1		157 (34.7)
2		160 (35.3)
3		49 (10.8)
4		16 (3.5)
5		0 (0)
SPPB total score	439	
Mean (SD)		9.64 (2.92)
Median (25th and 75th percentiles)		11.00 (8 and 12)
Low (0–6), no. (%)		56 (12.76)
Middle (7–9), no. (%)		96 (21.87)
High (10–12), no. (%)		287 (65.38)
MoCA total score	453	
Mean (SD)		24.37 (4.43)
Median (25th and 75th percentiles)		25 (22 and 27)
MCI of <26, no. (%)		284 (62.69)
Normal function, score of ≥26, no. (%)		169 (37.31)

^a^ BMI = body mass index; CVD = cardiovascular disease; MCI = mild cognitive impairment; MoCA = Montreal Cognitive Assessment; NIHSS = National Institutes of Health Stroke Scale; SPPB = Short Physical Performance Battery.

The study sample comprised 193 women (42.6%) and 260 men (57.4%), with a median age of 73.7 (25th and 75th percentiles = 65.8 and 80.4, respectively) years, mild to moderate symptoms at baseline (77.5% with a total score of <5 on the NIHSS), and mild levels of dependency at the 3-month follow-up point (85.5% with a score of <3 on the modified Rankin Scale). Global cognitive function of the study participants was estimated to be a median MoCA total score of 25 (25th and 75th percentiles = 22 and 27, respectively). Physical capacity was rated as middle to high in 87.3% of participants (median = 11; 25th and 75th percentiles = 8 and 12, respectively).

### Physical Activity Behavior After Stroke

Three months after stroke, 69.3% of the study population achieved the minimum threshold of 150 minutes of moderate-intensity activity per week, as recommended by the WHO.^41^ The accelerometric estimates of PA behavior within the study sample can be seen in Table 2. The mean recording time was 5.8 (SD = 0.6) days.

**Table 2:** Physical Activity Behavior Three Months After Stroke (N = 453)*^a^*

**Physical Activity Behavior/wk**	**Value**
Time spent upright, min, mean (SD)	1831.80 (787.22)
LPA walking, min, mean (SD)	233.16 (127.39)
MPA walking, min, mean (SD)	251.66 (164.62)
Short-bout MPA, min, mean (SD)	234.00 (145.23)
Any MPA participation, no. (%)	451 (99.6)
Long-bout MPA, min, mean (SD)	16.84 (48.60)
Long-bout MPA participation, no. (%)	106 (23.4)
No. of walking bouts, mean (SD)	1374.69 (691.61)
Bout length, walking, s/bout, mean (SD)	20.90 (7.28)
No. of sit-to-stand transitions, mean (SD)	311.36 (100.51)
Bout length, upright, min/bout, mean (SD)	6.14 (2.96)
WHO adherence, no. (%)*^b^*	314 (69.3)

^a^ LPA = light physical activity; MPA = moderate physical activity.

^b^ Adherence to the physical activity guidelines of the World Health Organization (WHO), defined as accumulating ≥150 min of MPA/wk.

**Figure 2:**
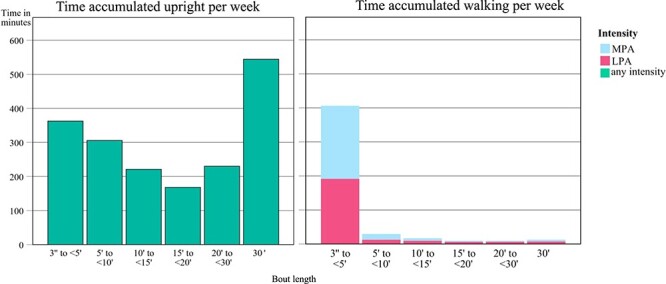
Physical activity (PA) behavior after stroke. Walking is presented as light physical activity (LPA) and moderate physical activity (MPA), both following a similar pattern of a substantial decrease in physical activity participation with increasing bout length (right graph). Time spent upright is presented in the left graph, comprising both standing and walking events of any intensity.

Time spent walking mostly occurred in short activity bouts lasting <10 minutes (93.3% of the time); the mean bout length for walking was 20.9 (SD = 7.28) seconds. Almost all participants performed at least 1 bout of MPA (99.6%), while participation in long-bout MPA was observed in less than a quarter of the group (23.4%). Upright time (walking and standing) was found to be distributed over all bout-length zones (Fig. 2), with most of the time concentrated in bouts of at least 30 minutes—indicating an activity pattern with primarily short walking bouts and many standing breaks. On average, light-intensity walking and moderate-intensity walking accounted for 233.2 (SD = 127.39) minutes and 251.7 (SD = 164.6) minutes of total upright time, respectively. Vigorous PA was not registered in any participant. High SDs were observed for all PA variables. Further details regarding the accumulation pattern of physical activity three months after stroke can be found in Suppl. Table 2.

### Association of Global Cognitive Function, Moderate PA, and Adherence to WHO Recommendations

**Figure 3:**
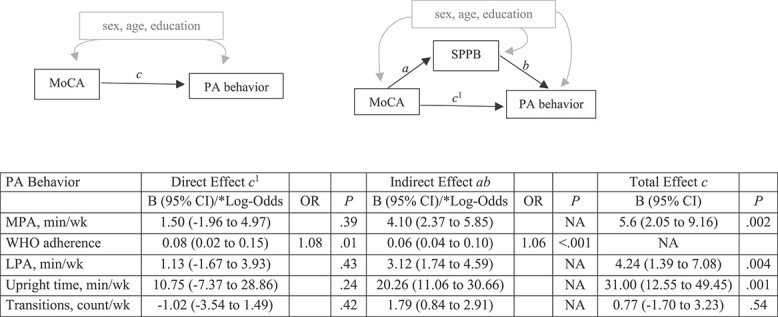
Direct, indirect, and total effects of Montreal Cognitive Assessment (MoCA) and physical activity (PA) behavior after stroke. (I) Physical capacity (Short Physical Performance Battery [SPPB]) is visualized as a mediator variable of the association of MoCA and PA behavior after stroke. The total effect *c* represents the association of MoCA and PA behavior after adjustment for sex, age, and education. The total effect *c* can be explained through direct (*c*^1^) and indirect (*ab*) effects of exposure and the outcome variable. The indirect effect *ab* describes how MoCA is linked to physical capacity (SPPB) which, in turn, influences PA behavior. The sample size in the mediation analysis model was 439 participants. (II) Direct, indirect, and total effects are presented as nonstandardized regression coefficients (B) for all continuous variables. *Outcomes for the bivariate variable adherence to PA recommendations of the World Health Organization (WHO) (accumulating at least 150 min of moderate to vigorous PA/wk) are expressed in log-odds metrics. The total effect *c* has not been estimated here because, for dichotomous outcome variables, *c* usually will not equal *c*^1^ + *ab*.^42^ LPA = light PA; MPA = moderate PA; NA = not applicable; OR = odds ratio; upright = time accumulated in standing or walking behavior at any intensity/nonsedentary; transitions = number of sit-to-stand transitions.

The results of the multiple regression analyses are reported in Table 3. Higher MoCA scores were positively associated with greater total amount of MPA performed during a week. The results did not show an association of MoCA with the number of sit-to-stand transitions. The direct, indirect, and total effects of MoCA on PA behaviors are presented in Figure 3; the statistically significant degree of change observed for the PA behavior variables (MPA, LPA, upright time) for every 1 point of change in the total MoCA score (total effect *c*) was found to diminish after the inclusion of SPPB as a mediator in the mediation model (direct effect *c*^1^). A 1-point increase in the MoCA score was associated with 5.6 minutes more time spent in MPA per week (total effect *c*), of which 4.1 minutes were attributed to the mediating effect of SPPB (indirect effect *ab*).

**Table 3:** Regression Analyses With Physical Activity Behavior as Dependent Variable and MoCA, Sex, Age, and Education as Simultaneous Covariates*^a^*

Dependent Variable	Independent Variable	B	95% CI for B	OR	95% CI for OR	*P*
Time spent in MPA, walking**,** min/wk	MoCA	6.23	2.73–9.74			.001
	Sex, men	38.14	10.62–65.67			.01
	Age	−4.03	−5.38 to −2.68			<.001
	Education	6.14	2.14–10.14			.003
Time spent in LPA, walking, min/wk	MoCA	4.59	1.82–7.37			.001
	Sex, men	26.37	4.56–48.17			.02
	Age	−3.05	−4.12 to −1.98			<.001
	Education	3.12	−0.05 to 6.28			.05
Time spent upright, min/wk	MoCA	32.31	14.14–50.49			.001
	Sex, men	−68.30	−211.04 to 74.44			.35
	Age	−8.12	−15.17 to −1.16			.02
	Education	17.17	−3.55 to 37.89			.10
No. of sit-to stand transitions	MoCA	0.83	−1.56 to 3.22			.50
	Sex, men	7.96	−10.80 to 26.72			.41
	Age	−1.44	−2.36 to −0.52			.002
	Education	−1.83	−4.55 to 0.90			.19
WHO adherence	MoCA			1.14	1.08 to 1.20	<.001
	Sex, men			0.56	0.36 to 0.88	.01
	Age			0.97	0.95 to 0.99	.02
	Education			1.05	0.99 to 1.12	.13

^a^ Statistical analysis was done using multiple linear regression for all continuous outcome variables and logistic regression models for the dichotomous outcome variable WHO adherence (adherence to the physical activity guidelines of the World Health Organization [WHO], defined as accumulating ≥150 min of MPA/wk). B = regression coefficient; LPA = light physical activity; MoCA = Montreal Cognitive Assessment; MPA = moderate physical activity; OR = odds ratio.

The log-odds metrics of the regression coefficient B, describing the strength of the association of MoCA with adherence to WHO PA recommendations, were estimated to be 0.08 and 0.06 for the direct and indirect effects, respectively. The transformation into odds ratio (Suppl. Appendix) resulted in values of 1.086 for the direct effect and 1.065 for the indirect effect of the total MoCA score on adherence to WHO recommendations. With each 1-point decrease in the MoCA score, there was an expected 8.6% increase in the odds of nonadherence to WHO PA recommendations. Further details of all mediation analyses can be seen in the Supplemental Appendix.

## Discussion

This study showed that the PA behavior at 3 months after stroke was fragmented, characterized by very short bouts of walking at moderate intensity that were interrupted by frequent bouts of standing activity. We also found that the majority (69.3%) of the participants engaged in at least 150 minutes of MPA per week, as recommended by the updated version of WHO PA guidelines.

Lower global cognitive function was associated with lower PA levels and decreased odds of adhering to WHO recommendations. In this context, physical capacity was determined to be an important mediating variable that can contribute to explaining the mechanisms underlying the relationship between cognition and PA behavior.

The PA behavior identified in this study suggests that the incorporation of shorter bouts of PA into clinical recommendations is more achievable at 3 months after stroke; this lends support to the current guidelines of the WHO.^41^ However, despite emerging evidence that engaging in short-bout PA is associated with similar health-related outcomes compared to performing continuous PA,^41^^,^^43^ we should question whether encouraging participation in longer bouts of moderate to vigorous PA remains important for many individuals with regard to their activities of daily living and their participative goals. Our finding, that none of the participants in this study reached PA levels of vigorous intensity, may lead physical therapists and their patients to adjust their aims and home-training interventions to align with evidence-based care after stroke, which includes the integration of repetitive task-oriented training at higher intensities.^21^^,^^44^^,^^45^ The incorporation of high-intensity training interventions has recently been demonstrated to be beneficial for both physical and cognitive functioning.^46^

Our findings align with previous observations that also point to little participation in longer bouts of walking^47^ or high-intensity PA^17^^,^^19^^,^^48^ after stroke. Attempts to explain the rather fragmented pattern of PA refer to occurrence or increment of pain, motor weakness, and fear of falling in periods of prolonged upright time,^49^ but the pattern can also be ascribed to reduced cardiorespiratory fitness after stroke.^50^ Based on the observation that PA seems to increase across all domains in the first 3 months after stroke and then reach a plateau, it has been hypothesized that behavior patterns established during these immediate poststroke months may influence PA behavior in the long term.^19^ This argumentation is in line with previous research highlighting the 3-month mark as a transition point from a period of structural and functional changes associated with early poststroke stages to a stabilized trajectory of cognitive recovery in the long term.^51^ This may have important implications regarding treatment strategies and the appropriate timing of interventions.

The mediation models presented in this study provide clinicians and researchers with more understanding of the importance of cognition for PA behavior and provide insight into the underlying mechanisms beyond what has previously been put forth. As the statistically significant direct effects of MoCA and the PA variables (MPA, LPA, upright time) disappear when SPPB is included in the mediation models, it can be concluded that the association of global cognitive function with PA behavior is mediated through physical capacity. Global cognitive function may hence be understood as an important antecedent of physical capacity which, in turn, influences MPA, LPA, and upright time. Our finding that global cognitive function also has a statistically significant direct effect on following WHO PA recommendations, indicates that adherence to PA recommendations relies on a cognitive component, beyond what can be explained through the effect of physical capacity. Fini et al. hypothesized that cognitive function could reflect the ability to problem-solve with regard to barriers to PA, as well as the ability to understand the importance of PA for future health outcomes.^23^

An explanation of why physical capacity mediates the association of global cognitive function with PA behavior can draw on the well-established association of cognition with balance, visuospatial skills, and strength of the lower limbs.^10^^,^^25^^,^^52^ It has been suggested that the link between gait speed and subjective memory can be explained by common risk factors; namely cardiovascular disease, diabetes mellitus, abnormal cortisol profiles, low vitamin D levels, brain atrophy with decreased hippocampal volume, and increased deposition of β-amyloid in the brain.^53^ In addition, walking has been described as a highly cognitive performance that is dependent on the interplay between multiple higher systems.^24^

### Limitations

The multicenter study design, represented by the inclusion of both large hospitals in centralized areas of Norway and smaller hospitals in slightly more rural regions of the country, increases the external validity. While objective PA measurements were taken from a sizable and representative study sample of individuals with low to moderate dependency in daily living 3 months after stroke,^54^ it is important to acknowledge that our findings may not be applicable to those who are severely affected by the stroke. Furthermore, data were collected in Norway which is a high-income country that invests in public health initiatives to promote PA. Additionally, it is important to consider the highly complex and multidependent processes behind behavioral outcomes, the possibility of further confounding, mediating, and/or moderating factors, and the potential of reverse causalities. Adherence to WHO PA recommendations in nearly 70% of the study population indicates that the odds ratio metrics might have been overestimated.^55^ There is a risk of overestimation of standing time together with an underestimation of walking time at light intensity. Although activPAL has almost perfect correlation and excellent agreement with direct observation for upright time, it has been found that its accuracy in detecting stepping is limited at very slow walking speeds.^38^^,^^39^ It should be noted that PA measurements were delimited to standing and walking activities, which are measurable by activPAL; hence, other activities (eg, strength training or static high intensity movements in a sitting position) were not accounted for. This may have led to a misclassification of PA to sedentary domains. Consideration should also be given to the use of absolute energy expenditure (in METs) based on the walking cadence. Because of naturally occurring fluctuations in stepping speeds while walking (eg, walking in the home, crowded places, traffic lights), an accurate estimate of bout length by intensity could not be determined. However, the approach of examining each walking bout individually, considering intrabout changes by presenting the percentage of moderate and light walking per bout (as done in this study), has recently been substantiated as more accurate^56^ than previously used approaches that estimate walking intensity per bout by calculating the average per time interval.^38^ The differences between included and not-included participants in this study indicate a propensity among the not-included participants of having higher age and living alone. Lastly, since participants couldn’t be blinded to study outcomes, we cannot rule out the possibility that the presence of accelerometer reactivity influenced PA behavior during the measurement period.^57^ Yet, there is currently not enough evidence to support the use of activity monitors as an intervention to increase PA after stroke.^58^

It can be concluded that highly individual PA patterns observed in this study underscore the need for personalized rehabilitation after stroke. Stroke survivors with reduced cognitive function are at higher risk of inactivity early after their stroke. Our findings suggest that interventions targeting good cognitive function after stroke may be important for improving adherence to PA guidelines among stroke survivors and increasing participation in MPA, LPA, and upright time by enhancing physical capacity. In future, clinical trials will have to identify the most effective ways of promoting PA among individuals with cognitive impairment after stroke. Whether specific domains of cognitive function are differently associated with PA behavior after stroke and whether they follow different mechanisms will have to be explored in more advanced models.

## Supplementary Material

PTJ-2022-0325_R2_Supplemental_Appendix_pzad092

## Data Availability

The MATLAB code and data of this study are available from the corresponding author upon reasonable request.

## Clinical Trial

The Nor-COAST study was registered in Clinicaltrials.gov (NCT02650531).
