# Supplementary material for: Physical Activity Behavior and Its Association With Global Cognitive Function Three Months After Stroke: The Nor-COAST Study
Source: Phys Ther. 2023 Jul 13;103(12):pzad092. doi: 10.1093/ptj/pzad092 (PMC10733132; doi:10.1093/ptj/pzad092)
Supplement: PTJ-2022-0325_R2_Supplemental_Appendix_pzad092 [file ptj-2022-0325_r2_supplemental_appendix_pzad092.pdf]

Supplemental Table 1. Baseline characteristics of included and not included participants tested after 3 months

Characteristic	Included n=453	Excluded n=247	p-value
**Age, y; mean (SD)**	72.49 (11.31)	74.99 (12.55)	
**Median, (25th, 75th percentile)**	**73.65 (65.83, 80.39)**	**77.11 (67.74, 84.30)**	**0.004**
Sex, male; n (%)	260 (57.4)	139 (56.3)	0.81
Education, y; mean (SD)	12.43 (3.64)	11.78 (3.91)	0.03
Living situation			0.001
alone, n (%)	143 (31.6)	91(36.8)	
with someone, n (%)	310 (68.4)	150 (60.7)	
institution, n (%)	0 (0)	6 (2.4)	
BMI, kg/m^2; mean (SD),	26.14 (4.25)	25.56 (4.07)	0.66
Missing, n (%)		17 (0.1)	
Diabetes, n (%)	81(17.9)	29(11.7)	0.03
Missing, n (%)	6 (1.3)	1 (0.4)	
Prior CVD, n (%)	99 (21.9)	66 (26.7)	0.16
NIHSS score			0.46
<3, n (%)	225 (49.7)	116 (47.0)	
3-5, n (%)	126 (27.8)	64 (25.9)	
6-10, n (%)	51 (11.3)	32 (13.0)	
>10, n (%)	40 (8.8)	30 (12.1)	
Missing, n (%)	11 (2.4)	5 (2.0)	

Abbreviations: BMI=Body Mass Index; NIHSS=National Institutes of Health Stroke Scale. P-values have been estimated using *t*-test and Chi-square test where applicable.

Supplemental Table 2. Accumulation pattern of physical activity three months after stroke

Parameter (N=453)	Time/week by bout-length, mean (SD) in min/week						
	Zone 1	Zone 2	Zone 3	Zone 4	Zone 5	Zone 6	Total
Time upright	362.60 (130.21)	305.59 (120.98)	221.17 (108.42)	168.18 (104.02)	230.12 (155.56)	544.08 (515.65)	1831.75 (787.22)
Time walking							
LPA	191.51 (102.77)	12.46 (13.21)	9.41 (14.18)	6.30 (12.36)	6.33 (14.95)	7.16 (21.21)	233.16 (127.39)
MPA	214.88 (128.58)	17.02 (30.95)	7.50 (19.86)	3.47 (11.09)	3.28 (11.48)	5.51 (27.21)	251.66 (164.62)

All outcome values are presents as population mean (SD). The zones 1 to 6 are defined by bout length per activity event, where applicable. Abbreviations: PA=physical activity; LPA=light physical activity; MPA=moderate physical activity; Zone 1=3 sec to <5 min; Zone 2=5 minutes to <10 minutes; Zone 3=10 minutes to <15minutes; Zone 4=15 minutes to <20 minutes; Zone 5=20 minutes to <30 minutes; Zone 6 ≥ 30 minutes.

Supplemental Appendix

Mediation Analyses, PROCESS Procedure for SPSS Version 4.0

```
         Written by Andrew F. Hayes, Ph.D.       www.afhayes.com
    Documentation available in Hayes (2022). www.guilford.com/p/hayes3

Level of confidence for all confidence intervals (CI) in output: 95,0000

Number of bootstrap samples for percentile bootstrap CI:5000

**************************************************************************
```

Moderate physical activity

```
**************************************************************************

    Y: MPA
    X: MoCA
    M: SPPB

Covariates: age sex  education                     Sample Size:  439
**************************************************************************
OUTCOME VARIABLE: SPPB

Model Summary
          R       R-sq        MSE          F        df1        df2          p
      ,5138      ,2640     6,3486    38,9104     4,0000   434,0000      ,0000

Model
              coeff         se          t          p       LLCI       ULCI
constant     6,7660     1,4803     4,5707      ,0000     3,8566     9,6755
MoCA          ,1979      ,0316     6,2716      ,0000      ,1359      ,2599
age         -,0569      ,0120    -4,7448      ,0000     -,0805     -,0333
sex           ,6811      ,2461     2,7680      ,0059      ,1975     1,1648
educ          ,0878      ,0357     2,4628      ,0142      ,0177      ,1579

**************************************************************************
OUTCOME VARIABLE: MPA

Model Summary
          R       R-sq        MSE          F        df1        df2          p
      ,5711      ,3261 18159,1657    41,9107     5,0000   433,0000      ,0000

Model
              coeff         se          t          p       LLCI       ULCI
constant   140,1098    81,0521     1,7286      ,0846   -19,1946   299,4143
MoCA         1,5014     1,7623      ,8520      ,3947    -1,9623     4,9650
SPPB        20,7352     2,5672     8,0769      ,0000    15,6894    25,7810
age         -2,9595      ,6579    -4,4987      ,0000    -4,2525    -1,6665
sex         18,5036    13,2767     1,3937      ,1641    -7,5913    44,5984
educ         4,9622     1,9200     2,5845      ,0101     1,1885     8,7358

************************** TOTAL EFFECT MODEL ****************************
OUTCOME VARIABLE: MPA

Model Summary
          R       R-sq        MSE          F        df1        df2          p
      ,4739      ,2246 20846,9097    31,4276     4,0000   434,0000      ,0000
```

Model

	coeff	se	t	p	LLCI	ULCI
constant	280,4050	84,8258	3,3057	,0010	113,6845	447,1254
MoCA	5,6044	1,8080	3,0998	,0021	2,0509	9,1580
age	-4,1395	,6873	-6,0231	,0000	-5,4902	-2,7887
sex	32,6273	14,1014	2,3138	,0211	4,9117	60,3430
educ	6,7828	2,0430	3,3201	,0010	2,7674	10,7981

************** TOTAL, DIRECT, AND INDIRECT EFFECTS OF X ON Y **************

Total effect of X on Y

Effect	se	t	p	LLCI	ULCI
5,6044	1,8080	3,0998	,0021	2,0509	9,1580

Direct effect of X on Y

Effect	se	t	p	LLCI	ULCI
1,5014	1,7623	,8520	,3947	-1,9623	4,9650

Indirect effect(s) of X on Y:

	Effect	BootSE	BootLLCI	BootULCI
SPPB	4,1031	,8969	2,3078	5,8920

Light physical activity

```
**************************************************************************
    Y: LPAwalk
    X: MoCA
    M: SPPB

Covariates: age    sex   educ                         Sample Size:  439

**************************************************************************
OUTCOME VARIABLE: SPPB

Model Summary
          R       R-sq        MSE          F        df1        df2          p
      ,5138      ,2640     6,3486    38,9104     4,0000   434,0000      ,0000

Model
              coeff         se          t          p       LLCI       ULCI
constant     6,7660     1,4803     4,5707      ,0000     3,8566     9,6755
MoCA          ,1979      ,0316     6,2716      ,0000      ,1359      ,2599
age         -,0569      ,0120    -4,7448      ,0000     -,0805     -,0333
sex          ,6811      ,2461     2,7680      ,0059      ,1975     1,1648
educ          ,0878      ,0357     2,4628      ,0142      ,0177      ,1579

**************************************************************************
OUTCOME VARIABLE: LPAwalk

Model Summary
          R       R-sq        MSE          F        df1        df2          p
      ,5300      ,2809 11834,2282    33,8200     5,0000   433,0000      ,0000

Model
              coeff         se          t          p       LLCI       ULCI
constant   172,9685    65,4314     2,6435      ,0085    44,3659   301,5712
MoCA         1,1302     1,4226      ,7945      ,4274    -1,6659     3,9263
SPPB        15,6956     2,0725     7,5734      ,0000    11,6223    19,7689
age         -2,2606      ,5311    -4,2567      ,0000    -3,3044    -1,2168
sex         13,4262    10,7180     1,2527      ,2110    -7,6396    34,4919
educ         2,0165     1,5500     1,3010      ,1939    -1,0299     5,0630

************************** TOTAL EFFECT MODEL ****************************
OUTCOME VARIABLE: LPAwalk

Model Summary
          R       R-sq        MSE          F        df1        df2          p
      ,4308      ,1856 13370,9569    24,7251     4,0000   434,0000      ,0000

Model
              coeff         se          t          p       LLCI       ULCI
constant   279,1655    67,9342     4,1094      ,0000   145,6446   412,6864
MoCA         4,2361     1,4480     2,9255      ,0036     1,3902     7,0820
age         -3,1538      ,5504    -5,7299      ,0000    -4,2355    -2,0720
sex         24,1172    11,2934     2,1355      ,0333     1,9207    46,3137
educ         3,3947     1,6361     2,0748      ,0386      ,1789     6,6104

************** TOTAL, DIRECT, AND INDIRECT EFFECTS OF X ON Y **************

Total effect of X on Y
     Effect         se          t          p       LLCI       ULCI
     4,2361     1,4480     2,9255      ,0036     1,3902     7,0820

Direct effect of X on Y
     Effect         se          t          p       LLCI       ULCI
```

```
    1,1302     1,4226      ,7945      ,4274    -1,6659     3,9263

Indirect effect(s) of X on Y:
             Effect     BootSE   BootLLCI   BootULCI
SPPB         3,1058      ,7156     1,7428     4,5912
```

Total time upright

```
**************************************************************************
    Y: Upright
    X: MoCA
    M: SPPB

Covariates: age sex  educ                        Sample Size:  439

**************************************************************************
OUTCOME VARIABLE: SPPB

Model Summary
          R       R-sq        MSE          F        df1        df2          p
      ,5138      ,2640     6,3486    38,9104     4,0000   434,0000      ,0000

Model
              coeff         se          t          p       LLCI       ULCI
constant     6,7660     1,4803     4,5707      ,0000     3,8566     9,6755
MoCA          ,1979      ,0316     6,2716      ,0000      ,1359      ,2599
age          -,0569      ,0120    -4,7448      ,0000     -,0805     -,0333
sex          ,6811      ,2461     2,7680      ,0059      ,1975     1,1648
educ          ,0878      ,0357     2,4628      ,0142      ,0177      ,1579

**************************************************************************
OUTCOME VARIABLE: Upright

Model Summary
          R       R-sq        MSE          F        df1        df2          p
      ,4489      ,2015 496528,641    21,8561     5,0000   433,0000      ,0000

Model
              coeff         se          t          p       LLCI       ULCI
constant   982,3982   423,8268     2,3179      ,0209   149,3844  1815,4120
MoCA        10,7459     9,2149     1,1661      ,2442    -7,3656    28,8575
SPPB       102,3760    13,4242     7,6262      ,0000    75,9914   128,7607
age         -3,7114     3,4400    -1,0789      ,2812   -10,4726     3,0497
sex       -156,2474    69,4248    -2,2506      ,0249  -292,6990   -19,7958
educ         9,8033    10,0398      ,9764      ,3294    -9,9295    29,5362

************************** TOTAL EFFECT MODEL ****************************
OUTCOME VARIABLE: Upright

Model Summary
          R       R-sq        MSE          F        df1        df2          p
      ,3070      ,0943 561923,589    11,2928     4,0000   434,0000      ,0000

Model
              coeff         se          t          p       LLCI       ULCI
constant  1675,0779   440,3984     3,8036      ,0002   809,4989  2540,6569
MoCA        31,0040     9,3868     3,3029      ,0010    12,5547    49,4534
age         -9,5372     3,5681    -2,6729      ,0078   -16,5502    -2,5243
sex        -86,5141    73,2119    -1,1817      ,2380  -230,4080    57,3798
educ        18,7922    10,6067     1,7717      ,0771    -2,0547    39,6390

************** TOTAL, DIRECT, AND INDIRECT EFFECTS OF X ON Y **************
```

Total effect of X on Y

Effect	se	t	p	LLCI	ULCI
31,0040	9,3868	3,3029	,0010	12,5547	49,4534

Direct effect of X on Y

Effect	se	t	p	LLCI	ULCI
10,7459	9,2149	1,1661	,2442	-7,3656	28,8575

Indirect effect(s) of X on Y:

	Effect	BootSE	BootLLCI	BootULCI
SPPB	20,2581	5,0099	11,0617	30,656

Short bout moderate physical activity

****

Y: shortMPA
X: MoCA
M: SPPB

Covariates: age sex education

Sample Size: 439

****

OUTCOME VARIABLE: SPPB

Model Summary

R	R-sq	MSE	F	df1	df2	p
,5138	,2640	6,3486	38,9104	4,0000	434,0000	,0000

Model

	coeff	se	t	p	LLCI	ULCI
constant	6,7660	1,4803	4,5707	,0000	3,8566	9,6755
MoCA	,1979	,0316	6,2716	,0000	,1359	,2599
age	-,0569	,0120	-4,7448	,0000	-,0805	-,0333
sex	,6811	,2461	2,7680	,0059	,1975	1,1648
education	,0878	,0357	2,4628	,0142	,0177	,1579

****

OUTCOME VARIABLE: shortMPA

Model Summary

R	R-sq	MSE	F	df1	df2	p
,5695	,3244	14075,9976	41,5788	5,0000	433,0000	,0000

Model

	coeff	se	t	p	LLCI	ULCI
constant	117,2808	71,3602	1,6435	,1010	-22,9746	257,5362
MoCA	1,8310	1,5515	1,1801	,2386	-1,2185	4,8804
SPPB	19,2021	2,2602	8,4956	,0000	14,7597	23,6445
age	-2,3941	,5792	-4,1335	,0000	-3,5325	-1,2557
sex	15,2672	11,6891	1,3061	,1922	-7,7073	38,2417
education	3,0685	1,6904	1,8152	,0702	-,2540	6,3909

****************** DIRECT AND INDIRECT EFFECTS OF X ON Y ****************

Direct effect of X on Y

Effect	se	t	p	LLCI	ULCI
1,8310	1,5515	1,1801	,2386	-1,2185	4,8804

Indirect effect(s) of X on Y:

	Effect	BootSE	BootLLCI	BootULCI

SPPB	3,7997	,8407	2,1452	5,4490

Long bout moderate physical activity

```
**************************************************************************
    Y: LongMPA
    X: MoCA
    M: SPPB

Covariates: age   sex  education

Sample Size:  439

**************************************************************************
OUTCOME VARIABLE: SPPB

Model Summary
          R       R-sq        MSE          F        df1        df2          p
      ,5138      ,2640     6,3486    38,9104     4,0000   434,0000      ,0000

Model
              coeff         se          t          p       LLCI       ULCI
constant     6,7660     1,4803     4,5707      ,0000     3,8566     9,6755
MoCA          ,1979      ,0316     6,2716      ,0000      ,1359      ,2599
age         -,0569      ,0120    -4,7448      ,0000     -,0805     -,0333
sex           ,6811      ,2461     2,7680      ,0059      ,1975     1,1648
education     ,0878      ,0357     2,4628      ,0142      ,0177      ,1579

**************************************************************************
OUTCOME VARIABLE: LongMPA

Model Summary
          R       R-sq        MSE          F        df1        df2          p
      ,2601      ,0676  2287,8885     6,2833     5,0000   433,0000      ,0000

Model
              coeff         se          t          p       LLCI       ULCI
constant    22,8290    28,7696      ,7935      ,4279   -33,7164    79,3744
MoCA        -,3296      ,6255     -,5269      ,5985    -1,5590      ,8998
SPPB         1,5331      ,9112     1,6825      ,0932     -,2579     3,3241
age         -,5654      ,2335    -2,4213      ,0159    -1,0243     -,1065
sex          3,2364     4,7126      ,6868      ,4926    -6,0260    12,4988
education    1,8937      ,6815     2,7787      ,0057      ,5542     3,2332

***************** DIRECT AND INDIRECT EFFECTS OF X ON Y ****************

Direct effect of X on Y
     Effect         se          t          p       LLCI       ULCI
     -,3296      ,6255     -,5269      ,5985    -1,5590      ,8998

Indirect effect(s) of X on Y:
             Effect     BootSE   BootLLCI   BootULCI
SPPB          ,3034      ,1060      ,1175      ,5326
```

Number of sit-to-stand transitions

```
**************************************************************************
    Y: Transit
    X: MoCA
    M: SPPB

Covariates: age sex   educ                                Sample Size:  439

**************************************************************************
OUTCOME VARIABLE: SPPB
Model Summary
          R       R-sq        MSE          F        df1        df2          p
      ,5138      ,2640     6,3486    38,9104     4,0000   434,0000      ,0000

Model
              coeff         se          t          p       LLCI       ULCI
constant     6,7660     1,4803     4,5707      ,0000     3,8566     9,6755
MoCA          ,1979      ,0316     6,2716      ,0000      ,1359      ,2599
age          -,0569      ,0120    -4,7448      ,0000     -,0805     -,0333
sex          ,6811      ,2461     2,7680      ,0059      ,1975     1,1648
educ          ,0878      ,0357     2,4628      ,0142      ,0177      ,1579

**************************************************************************
OUTCOME VARIABLE: Transit

Model Summary
          R       R-sq        MSE          F        df1        df2          p
      ,2811      ,0790  9582,8983     7,4274     5,0000   433,0000      ,0000

Model
              coeff         se          t          p       LLCI       ULCI
constant   339,1470    58,8796     5,7600      ,0000   223,4216   454,8723
MoCA        -1,0245     1,2802     -,8003      ,4240    -3,5407     1,4916
SPPB         9,0562     1,8649     4,8560      ,0000     5,3907    12,7216
age          -,8722      ,4779    -1,8251      ,0687    -1,8115      ,0671
sex          1,8383     9,6448      ,1906      ,8489   -17,1181    20,7947
educ        -2,3859     1,3948    -1,7106      ,0879    -5,1272      ,3555

************************** TOTAL EFFECT MODEL ****************************
OUTCOME VARIABLE: Transit

Model Summary
          R       R-sq        MSE          F        df1        df2          p
      ,1698      ,0288 10081,4950     3,2214     4,0000   434,0000      ,0127

Model
              coeff         se          t          p       LLCI       ULCI
constant   400,4213    58,9888     6,7881      ,0000   284,4820   516,3606
MoCA          ,7675     1,2573      ,6104      ,5419    -1,7037     3,2387
age         -1,3875      ,4779    -2,9032      ,0039    -2,3269     -,4482
sex          8,0069     9,8063      ,8165      ,4147   -11,2669    27,2806
educ        -1,5907     1,4207    -1,1197      ,2635    -4,3830     1,2016

************** TOTAL, DIRECT, AND INDIRECT EFFECTS OF X ON Y **************

Total effect of X on Y
     Effect         se          t          p       LLCI       ULCI
      ,7675     1,2573      ,6104      ,5419    -1,7037     3,2387

Direct effect of X on Y
     Effect         se          t          p       LLCI       ULCI
    -1,0245     1,2802     -,8003      ,4240    -3,5407     1,4916
```

```
Indirect effect(s) of X on Y:
             Effect     BootSE   BootLLCI   BootULCI
SPPB        1,7920      ,5308      ,8385     2,906
```

Logistic Regression in mediation analysis

Adherence to physical activity guidelines – WHO

```
**************************************************************************
    Y: Adh_WHO
    X: MoCA
    M: SPPB

Covariates: age   sex  education                       Sample Size:  439

**************************************************************************
OUTCOME VARIABLE: SPPB

Model Summary
          R       R-sq        MSE          F        df1        df2          p
      ,5138      ,2640     6,3486    38,9104     4,0000   434,0000      ,0000

Model
              coeff         se          t          p       LLCI       ULCI
constant     6,7660     1,4803     4,5707      ,0000     3,8566     9,6755
MoCA          ,1979      ,0316     6,2716      ,0000      ,1359      ,2599
age          -,0569      ,0120    -4,7448      ,0000     -,0805     -,0333
sex           ,6811      ,2461     2,7680      ,0059      ,1975     1,1648
education     ,0878      ,0357     2,4628      ,0142      ,0177      ,1579

**************************************************************************
OUTCOME VARIABLE: Adh_WHO

Coding of binary Y for logistic regression analysis:
    Adh_WHO  Analysis
      ,00       ,00
     1,00      1,00

Model Summary
       -2LL    ModelLL         df          p   McFadden   CoxSnell   Nagelkrk
   416,8537   119,9926     5,0000      ,0000      ,2235      ,2392      ,3389

Model
              coeff         se          Z          p       LLCI       ULCI
constant    -4,0466     1,6630    -2,4332      ,0150    -7,3061     -,7870
MoCA          ,0827      ,0324     2,5477      ,0108      ,0191      ,1462
SPPB          ,3195      ,0503     6,3586      ,0000      ,2210      ,4180
age          -,0150      ,0135    -1,1129      ,2658     -,0414      ,0114
sex           ,3587      ,2482     1,4454      ,1484     -,1277      ,8451
education     ,0395      ,0365     1,0819      ,2793     -,0321      ,1110

These results are expressed in a log-odds metric.

***************** DIRECT AND INDIRECT EFFECTS OF X ON Y ****************

Direct effect of X on Y
     Effect         se          Z          p       LLCI       ULCI
      ,0827      ,0324     2,5477      ,0108      ,0191      ,1462

Indirect effect(s) of X on Y:
```

	Effect	BootSE	BootLLCI	BootULCI
SPPB	,0632	,0164	,0351	,1002

SPPB	Effect	se	Z	p
	,0632	,0142	4,4374	,00000

*********************** Transformation in Odds Ratio *********************

Direct effect of X on Y: $e^{0.0827} = 1.0862158949$
Indirect effect of X on Y: $e^{0.0632} = 1.0652398659$

NOTE: Total effect model not available with dichotomous Y
NOTE: Direct and indirect effects of X on Y are on a log-odds metric.

****

Written by Andrew F. Hayes, Ph.D. www.afhayes.com
Documentation available in Hayes (2022). www.guilford.com/p/hayes3
